# RETRACTION: “Differential Effects of AKT1(p.E17K) Expression on Human Mammary Luminal Epithelial and Myoepithelial Cells”

**DOI:** 10.1155/humu/9801731

**Published:** 2026-03-17

**Authors:** Human Mutation

RETRACTION: B. Salhia, C. Van Cott, T. Tegeler, A. Polpitiya, R. A. DuQuette, M. Gale, G. Hostteter, K. Petritis, and J. Carpten, “Differential Effects of AKT1(p.E17K) Expression on Human Mammary Luminal Epithelial and Myoepithelial Cells,” *Human Mutation* 33, no. 8 (2012): 1216–27, https://doi.org/10.1002/humu.22100.

The above article, published online on 13 April 2012 in Wiley Online Library (wileyonlinelibrary.com), has been retracted by agreement between the authors; the journal Assistant Editor, Priya Gusain; and Wiley Periodicals LLC. The retraction has been agreed due to concerns originally raised by *Zachria flavicoma* and *Mycosphaerella arachidis* via PubPeer [1], regarding unexpected similarities in the western blot bands of Figures 1 and 2.

During an investigation into these concerns, the authors stated that these discrepancies were likely due to errors during figure preparation but confirmed that the underlying data are no longer available. As such, the authors have agreed to the retraction due to concerns with the reliability of the data.
